# Stoma care nurses’ experiences using a clinical feedback system in routine outpatient follow-up: a qualitative study

**DOI:** 10.1186/s12912-026-05100-1

**Published:** 2026-07-22

**Authors:** Lill Anette Juvik, John Roger Andersen, Julia Kittscha, Kirsten Lerum Indrebø, Anne Marie Sandvoll

**Affiliations:** 1 Department of Surgery, Førde Hospital Trust, Svanehaugvegen 2, Førde, 6812 Norway; 2 Department of Health and Caring Sciences, Western Norway University of Applied Sciences, Førde, Norway; 3 Department of Research and Innovation, Førde Hospital Trust, Førde, Norway; 4 Division of Surgery, Illawarra Shoalhaven Local Health District, Wollongong Hospital, Wollongong, Australia; 5 University of Wollongong, Wollongong, Australia

**Keywords:** Ostomy care, Stoma care nurses, Clinical feedback system, Patient-reported outcomes, Qualitative research, Person-centred care

## Abstract

**Background:**

A clinical feedback system (CFS) has been implemented in routine outpatient follow-up to collect patient-reported outcomes (PROs), enabling stoma care nurses (SCNs) to address patients’ needs as they adjust to life with an ostomy. This study aimed to explore SCNs’ experiences of using a CFS in routine outpatient follow-up within specialist healthcare services.

**Methods:**

The study had an exploratory, inductive qualitative design. Semi-structured, individual interviews were conducted with fourteen SCNs at outpatient clinics in four hospitals. Data were analysed using Reflexive thematic analysis.

**Results:**

An overarching theme, *Clinical feedback system as a tool for personalised and holistic follow-up care*, was developed with three themes: (1) Providing and receiving information as preparation for consultation; (2) Seamless use of clinical feedback system during consultations; and (3) Shared language and use of visual features. SCNs experienced the use of the CFS as a valuable tool for promoting personalised and holistic follow-up care. They described the CFS as supporting preparation, providing structure to consultations, and enhancing patient involvement. The CFS was experienced as useful for mapping, assessing, and evaluating patients’ adaptation processes, particularly in identifying areas of concern. SCNs described using the tool seamlessly within their existing practice while exercising clinical judgement. They experienced the visual features and the development of a shared, mutually understood language as facilitating meaningful dialogue during consultations. Although perceptions of its usefulness varied, SCNs emphasised that the CFS allowed flexibility and autonomy, enabling them to adapt its use to their consultation style and patients’ individual needs.

**Conclusion:**

This study showed that SCNs experienced the CFS as more than a tool for assessing patient function and adaptation and described how it shaped the way they conducted follow-up consultations. The SCNs perceived that meaningful use in routine practice supported their work in tailoring holistic follow-up care to individual patients. These findings suggest that the use of a CFS in routine ostomy care has the potential to facilitate person-centred dialogue and support person-centred care. This study highlights an emerging, forward-looking nursing role in which digital tools are integrated into everyday practice to enhance personalised care. Developing structured training programmes for SCNs is essential to support the effective use of the CFS in clinical practice. Further research is warranted to inform the development, content, delivery, and effects of such programmes.

**Clinical trial number:**

Not applicable.

**Supplementary Information:**

The online version contains supplementary material available at https://doi.org/10.1186/s12912-026-05100-1.

## Background

Stoma care nurses (SCNs) are essential in supporting patients with an ostomy as they adapt to life with their condition [1]. Each year, approximately 3,700 individuals undergo ostomy surgery in Norway [2] due to cancer, inflammatory bowel disease, infections, congenital abnormalities, or other diagnoses [3]. Ostomy surgery involves rerouting a section of the intestine through the abdominal wall, allowing urine or faeces to pass into an external pouch, which requires physical and psychological adjustment [4]. These changes can profoundly affect physiological and psychosocial aspects of life [4–7] and overall quality of life [8]. Some may face challenges in adapting, such as managing leakage, performing stoma care, concerns about body image, and difficulties with sexuality or social interaction [5, 9]. However, a novel intervention using a clinical feedback system (CFS) with patient-reported outcomes (PROs) to address adjustment-related issues has catalysed this research [10].

Health authorities advocate using PROs, defined as “reports coming directly from patients about how they function or feel in relation to a health condition or intervention, without interpretation by anyone else” [11]. PROs are often utilised through CFSs, which employ standardised and validated patient-reported outcome measures (PROMs), i.e., tools designed to assess outcomes relevant to specific health conditions and completed by patients. The results are made available to healthcare professionals for assessment. Patient-reported data are increasingly integrated into clinical practice, with the primary goal of fostering patient-centred care and enabling patients to participate actively in decisions about their health [12]. The repeated use of PROMs throughout the care of the individual patient plays a crucial role in assessment, assists in clinical decision-making, tracks patients’ progress, and can influence the patient-clinician encounter [13].

A systematic review of the effectiveness of PROM use in clinical practice showed it yielded moderate improvements in patient-clinician communication and minor enhancements in quality of life [14]. Minor positive effects on treatment outcomes have been noted in mental healthcare [15]. Research has predominantly found positive outcomes, resulting in improved health-related quality of life, patient satisfaction, and enhanced patient-clinician communication in cancer care [16].

The perspectives of healthcare professionals on PROM use have been explored across various clinical settings, with reports of both benefits and limitations. The principal benefits include promoting active patient involvement, prioritising patient needs, improving care quality, monitoring standardised patient outcomes, and enhancing patient-clinician relationships [17]. Professionals value PROMs that support the clinical decision-making process [18]. The use of these measures is shaped by healthcare professionals’ relationships with patients and their professional roles and boundaries [19]. However, several limitations have been noted, including the potential for PROMs to shift the focus of consultations, give rise to unrealistic expectations for care, lack clinically meaningful information, or provide unsuitable patient information [17]. Significant barriers to using CFSs include a lack of time and knowledge to meaningfully interpret and integrate PRO data into clinical practice, and the inability to act on such data [20]. Major factors that may impede or promote adaptation in routine practice include PROM fit with existing practice, its value, its capacity to respond, and its available support [21].

As part of the specialist healthcare service, SCNs play a key role in routine outpatient follow-up after ostomy surgery [22]. A systematic review found that nursing interventions aimed at improving self-efficacy and adaptation in ostomy patients are critical and lead to a reduction in adverse patient outcomes [23]. However, there has been a paucity of intervention studies seeking to test ways to address adjustment-related issues [9]. A novel SCN-led intervention was developed using a CFS to collect PROs, allowing SCNs to review them to address patient needs more effectively during the adjustment process following ostomy surgery [10]. A subsequent quantitative study showed that patients were satisfied with their follow-up using pre-consultation questionnaires, received sufficient and individualised information, were involved in treatment decisions, and benefited from the consultations. Moreover, measures of daily activities, knowledge and skills, health, and health-related quality of life improved during the first year after surgery [24]. Results from qualitative studies of patients’ perspectives indicated that engaging with the CFS prior to consultations had personal utility across multiple dimensions, even though not all patients fully grasped its purpose. The CFS was found to prompt reflection and self-awareness and to function as a tool for communication [25]. During consultations, patients reported variations in how the SCNs used their responses. Nevertheless, patients found value in using the CFS, as it facilitated communication and provided a sense of reassurance when their health status was assessed. Regular use enabled patients to be more active in their care [26].

PROMs can support the practice of tailoring care to individual needs, aid clinical decision-making, and inform value-based healthcare initiatives [27, 28]. A recent study found that patients did not systematically bias their responses when they knew healthcare professionals would review their PROs, which supports the use of PROMs in clinical practice as a valid mechanism for promoting patient-centred care [29]. In ostomy care, effective communication between SCNs and patients regarding individual needs is essential [30]. Although existing research offers insight into healthcare professionals’ perspectives about using CFSs in various clinical settings, there is a lack of research on their application in ostomy care [24]. While the method appears promising from the patient’s perspective, potentially enabling more tailored adjustments to care, limited knowledge exists about how SCNs experience using a CFS to support patients’ adaptation and how its use influences clinical practice [25, 26]. Thus, this study aimed to explore the experiences of SCNs using a CFS in routine follow-up within the specialist healthcare service.

## Method

### Design

An exploratory, inductive qualitative design was employed, using semi-structured individual interviews. The reporting of this study’s results was based on standards for reporting qualitative research [31].

### Intervention and context

The Ostomy Adjustment System, a SCN-led CFS based on PROs, was developed at Førde Hospital Trust to improve the quality of consultations, enhance patient involvement, and support adaptation to life with an ostomy [10]. The development group included researchers, software developers, SCNs, and patient representatives The items were developed based on the needs of patients and SCNs and on clinical recommendations [22], and were standardised for the Norwegian population and evaluated in clinical development studies [24, 32–34]. The CFS consists of sociodemographic and clinical forms, the Ostomy Adjustment Scale (a six-point response scale that measures the adjustment process) and the Coop-Wonca Chart (a five-point Likert scale that measures health-related quality of life) [35, 36]. The CFS was first implemented at one outpatient clinic in 2017. As part of a predefined plan, Version 2, which consists of up to 86 items distributed across multiple scales, was implemented into routine follow-up care at three additional outpatient clinics in different hospitals within the Regional Health Authority in 2022. The implementation followed a bottom-up approach initiated by the SCNs and supported by management.

SCNs in Norway play a central role in the routine follow-up consultations with patients after ostomy surgery, which is part of the specialist healthcare service [22]. These consultations are guided by national professional recommendations, which schedule follow-up routinely at 3 weeks, 3 months, 6 months, and 12 months postoperatively, and then annually. These follow-up consultations include: (1) clinical control of the ostomy, skin, and ostomy equipment; and (2) discussions with the patient, including health education and guidance. SCNs also provide a low-threshold service for patients requiring more frequent follow-ups [22].

From three months after surgery onward, the CFS is incorporated into all routine follow-up consultations for patients aged 18 years or older, who can speak, read, and write Norwegian, and have a permanent urostomy, ileostomy, or colostomy. It is not used during low-threshold consultations. The CFS is administered electronically via smartphone, computer, or tablet, and the questionnaires are sent to patients 14 days before their follow-up consultations. The CFS generates visual reports with graphs and bars charts summarising patients’ adjustment and development over time. SCNs have access to each patient’s complete set of item responses. The reports become available to the SCNs immediately after the patient completes the questionnaire but are not available to the patients themselves. The patient and the SCN use the report collaboratively during the follow-up consultation.

**Fig. 1:**
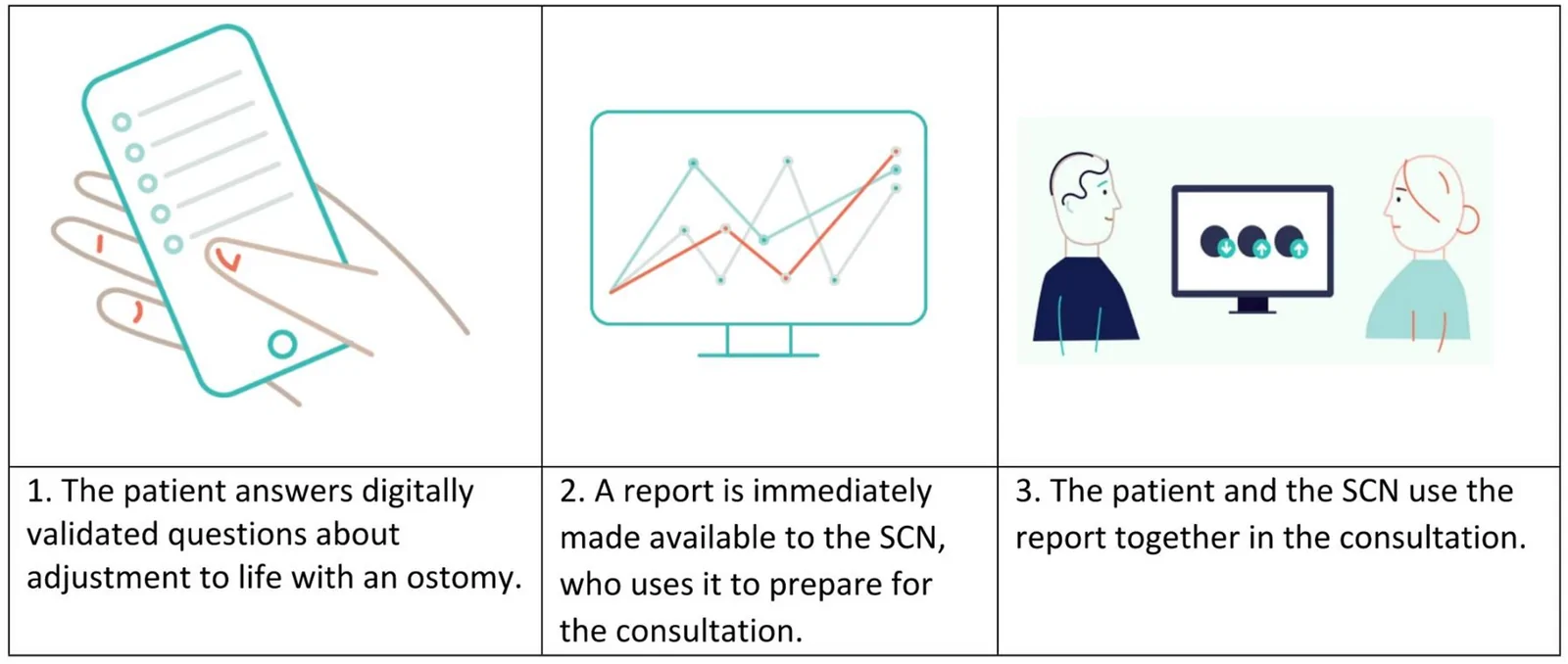
Use of the ostomy adjustment system

All SCNs received training in the use of the CFS, delivered by an SCN who from the intervention development team [10]. At each participating hospital, a designated SCN was responsible for coordinating with the intervention development team. The SCNs participated in joint training sessions covering PRO assessment and the use of the CFS. These sessions provided opportunities for professional discussion to facilitate a shared understanding and consistent use of the CFS across clinical settings. All participants had access to technical support. Responsibility for sending the questionnaires before consultations varied across hospitals and rested either with the SCN or the secretary. At the time of the study, 14 SCNs were employed across the four outpatient clinics. Figure 1 illustrates the use of the Ostomy Adjustment System.

### Participants and recruitment

A total population sampling strategy was used. Invitation emails containing information were sent to all SCNs employed at the four outpatient clinics in the hospitals where the CFS was implemented. All invited SCNs consented to participate and are hereafter referred to as SCNs or participants. All participants were women aged 28 to 65 years. Their outpatient clinic employment ranged from 15% to 100%, with the remainder of their positions allocated to inpatient wards or other responsibilities. Their length of employment in the outpatient clinics ranged from one to 27 years, with many having extensive experience in stoma care from previous work on inpatient wards. SCNs had experience from varying numbers of consultations in which patients had completed the questionnaire. SCNs had varying numbers of consultations in which patients completed the questionnaire. Those working in lower employment percentages generally had the least experience. One had no experience receiving PRO reports and had been on a period of leave. Detailed characteristics are presented in Table 1.

**Table 1:** Demographics of the stoma care nurse participants

Participant number	Age	Different hospitals within the Regional Health Authority	Number of consultations with patients answering questionnaires	Current position percentage in the outpatient clinic	Year(s) of experience in the outpatient stoma care clinic
P1	40s	1	50–99	60	6
P2	60s	1	≥ 100	40	24
P3	40s	1	50–99	40	4
P4	60s	2	10–49	100	3
P5	50s	2	50–99	50	19
P6	40s	4	4 ≤ 9	80	4
P7	60s	4	50–99	100	2
P8	60s	4	10–49	100	25
P9	50s	3	10–49	40	27
P10	50s	2	10–49	40	2
P11	40s	2	≤ 9	15	4
P12	60s	2	10–49	100	27
P13	20s	2	≤ 9	15	1
P14	20s	2	0 ≤ 9	15	1

Note: P6, P8, and P14 were on leave for periods after the clinical feedback system had been integrated into routine clinical practice

### Data collection

Three patient user representatives recruited by Førde Hospital Trust were involved in the reflection on and development of the interview guide. From October 2024 to November 2024, the first author conducted individual interview in a private room at the hospitals. One interview was conducted remotely via a secure video link. All interviews, which averaged 64 min (range = 45 to 136), were recorded. The interviews sought to elicit participants’ own practice narratives about their use of the CFS through an exploratory approach, with the interview guide serving as a flexible guide and support rather than a fixed sequence of questions while making transparent the topics identified in advance by the researchers (Supplementary Material 1). Initially, participants were asked about their professional background, their role in the development of the intervention, and their initial views of the CFS to provide contextual information for the subsequent conversation. Participants were then asked to select a patient they had followed up in the outpatient clinic and describe the consultation process and their clinical practice from the initial contact. A flowchart poster illustrating the stages of follow-up care and an example poster displaying PROs were placed on the table as visual prompts to support participants’ reflections (Supplementary Material 2 and 3). The interviewer followed participants’ accounts and the emerging lines of inquiry with open-ended, exploratory probing questions to encourage further elaboration and exploration. Participants were actively invited and challenged to reflect on experiences that differed from, challenged, or contrasted with their initial narratives. In some instances, the interviewer assumed the role of a patient and invited participants to demonstrate or describe how they would respond in different clinical situations to elicit more concrete and nuanced descriptions of clinical practice (e.g., " If I were that patient, how would you communicate this to me?“).

### Data analysis

A six-phase Reflexive thematic analysis was undertaken to explore patterns across the dataset [37]. This six-phase process included: (1) familiarisation with the dataset; (2) thematic coding; (3) development; (4) review; and (5) refinement of themes before (6) producing the final report. This approach was theoretically flexible, with the researcher’s subjectivity being integral to the analysis, viewing coding as an organic and flexible process that required detailed engagement with the data [37]. The first author analysed the data in close collaboration with the last author to seek new insights. The results section, including quotes presented throughout the themes, was initially written in the Norwegian before being translated into English. The quotations were drawn from the majority of participants. See Supplementary Material 4 for a detailed description of the analytical process.

### Ethical considerations

This study was conducted in accordance with the principles of the Declaration of Helsinki [38], and the local data protection officers approved the study at each hospital trust (reference number 5056). All participants received written information about the study’s purpose, anonymity, confidentiality, data security, and their right to withdraw at any time without consequences. Written informed consent was obtained from all participants prior to the interviews. Anonymised transcripts were stored on secure servers. To protect participants’ anonymity, quotations are not attributed to individual participants.

### Reflexivity

Reflexivity involves critical reflection on our roles as researchers and on the practices and processes employed [37]. LAJ, a PhD candidate, is a trained therapist and psychiatric nurse with experience in qualitative research. AMS is a Professor of Nursing Science with extensive experience in qualitative research. JK and KLI are practising SCNs holding PhDs in stoma care nursing. JRA is a Professor of Nursing Science. All authors critically reflected on their preconceptions and interests and approached the data and the developing findings with an open and inquisitive attitude. LAJ, AMS, and JK were not involved in the development of the intervention. During the study, LAJ was employed by Førde Hospital Trust as a PhD candidate. The first author engaged closely with the dataset over an extended period, allowing analytical insights to develop. The analytical work was continuously challenged and discussed with co-authors and researchers outside the immediate research environment to establish meta-positions and identify blind spots. A reflexive research diary was maintained throughout the analytical process to strengthen the quality of the analysis. Among other reflections, the first author’s initial assumptions about the expected findings were revisited as a reflexive strategy to examine the extent to which these assumptions were confirmed or challenged by the empirical material. The potential influence of the first author’s relationship with the developers of the intervention on the research process was an ongoing topic of reflexive discussion with the supervisors throughout the study [37, 39].

## Results

An overarching theme, *Clinical feedback system as a tool for personalised and holistic follow-up care*, was developed through our analysis. This overarching theme provides contextual information for three interrelated themes concerning SCNs’ experiences of using the CFS in clinical practice: (1) Providing and receiving information as preparation for consultation; (2) Seamless use of clinical feedback system during consultations; and (3) Shared language and the use of visual features.

Reflecting on their clinical practice, the SCNs described various aspects of using the CFS, both before and during outpatient follow-up consultations. They emphasised that a trusting and positive relationship with the patient, combined with strong professional competence, was fundamental to providing high-quality care. At the same time, many experienced enhanced patient involvement and recognised the CFS as a valuable tool for providing holistic care tailored to patients’ needs.

The system introduced a new structure for consultation preparation and provided structure throughout the consultation. SCNs experienced the CFS as useful in mapping, assessing, and evaluating patients’ adaptation processes, particularly in identifying areas of concern. It was also perceived as a communication tool that helped ensure that important topics were addressed.

Many SCNs experienced that the CFS had become incorporated into their clinical practice, although its perceived utility varied. They found that the tool allowed for autonomy and flexibility in its use, enabling them to adapt it to their preferences and consultation style to support patients’ adjustment in the best possible way. Clinical judgement played a prominent role in utilising the CFS to prepare for and conduct consultations. A few experienced a lack of digital skills that limited their ability to use the CFS to its full potential. Several SCNs also pointed to shortcomings in quality assurance that affected patients’ access to the questionnaires.

SCNs experienced that mastery and effective use of the CFS developed through practical hands-on experience. Some believed the CFS could be useful as a support tool for less experienced SCNs. SCNs were open to different approaches to using the tool, recognising that variation in its use could generate valuable insights worth sharing within the professional community. A clear need was also expressed for further guidance to continue developing their competencies and to expand their repertoire of strategies for using the CFS in patient encounters.

The SCNs emphasised that PROs required further exploration to have clinical value and that the CFS could not replace individual clinical assessments. Some expressed scepticism towards narratives in public discourse suggesting that standardisation and the use of the CFS could reduce the need for healthcare services. They strongly advocated for the continued need for stoma care nursing expertise. Nevertheless, there was a shared perception that the CFS should be further implemented and integrated into clinical practice.

There was no uniform experience of using the CFS in clinical practice; however, the SCNs’ accounts revealed patterns that converged around this overarching theme and across the following three themes.

### Providing and receiving information as preparation for consultation

Using PROs introduced a new approach to consultation preparation for the SCNs. They experienced that ensuring patients were well informed was essential to obtaining meaningful feedback. During the three-week follow-up consultation and as part of the new intervention, they informed and prepared patients for using the CFS in ongoing follow-ups.

SCNs conveyed information about using the CFS in various ways without a standardised approach. Most followed routines for informing patients, though this was sometimes omitted. Some saw it as their responsibility to provide tailored information and ensure that patients understood how to use the CFS. Several emphasised the importance of clarity and ensuring patients’ understanding of the CFS’s purpose. One SCN gave an example of how she introduced the CFS to patients:

*These are questions about how you experience living with an ostomy – how you feel physically and mentally. You should answer based on your own experience. The answers should only be correct for you. This can help you prepare for the consultation – you might know what you want to ask and talk about. I will prepare by reading your answers before we meet […] How does that sound to you?*

The SCNs placed varying degrees of emphasis on the content of the information they conveyed. Some highlighted patient benefits, while others emphasised the advantages for stoma care nursing practice. Some expressed themselves more concisely, focusing on specific aspects of the method, such as its potential to support preparation, enhance consultations, and strengthen patient involvement. Others provided more comprehensive information, covering multiple topics and including the use of data for research purposes.

Participants expanded their preparation routines by incorporating feedback outcomes alongside patient records to guide consultations. They shared an understanding that the responses could not be interpreted in isolation. Professional assessments of the reports provided valuable insights into the patient’s adaptation process and potential challenges, thereby providing a valuable foundation for the consultation.

The preparation time varied; it was common practice to complete preparation on the day before or the same day, depending on how SCNs organised their workday and the organisational framework of each hospital. In general, they perceived the preparation time required for using the CFS as acceptable, and the process became more efficient with practice and increased familiarity with the database and instruments. When SCNs were already familiar with the patient’s situation, they sometimes retrieved information well in advance to assess whether it was necessary to consult other healthcare professionals.

*Sometimes*,* I checked whether any patients had submitted their responses just to get a sense of whether I should try to speak with the surgeon about something before the consultation.*

SCNs systematically reviewed the reports from the various feedback instruments (questionnaires/forms) to gain an overview. They assessed total scores and reviewed individual responses corresponding with unfavourable scores to develop a more detailed understanding of the patient’s situation.

*I go through the responses*,* and they’re easy to navigate. First*,* I review the clinical database and take note of anything with low scores. Then*,* I look at the OAS scale [Ostomy Adjustment Scale] and focus on high scores. I click on individual questions to see what they’re specifically about*,* and I do the same with the quality-of-life form. Ultimately*,* I develop an idea of what we must address in the consultation.*

While reviewing the patient’s responses, SCNs identified areas they wished to explore further in subsequent consultations. Particular attention was given to symptom alerts related to leakage and pain around the stoma, as well as the patient’s knowledge of hernia risks. The SCNs noted that patients may have different perceptions of what leakage entails, and they paid particular attention to this during the follow-up consultation. They focused on all domains of the Ostomy Adjustment Scale, as these offered valuable insight into the patient’s adaptation process. Experiences with the quality-of-life form were mixed. Although it often provided useful information, the participants observed that unfavourable scores were not always related to stoma-specific issues. Other health conditions or life circumstances could influence the responses, necessitating further exploration with the patient. Several participants also found that they could interpret the responses in light of outcomes from the other forms and consider information such as the patient’s diagnosis documented in the medical record. When inconsistencies emerged, they explored possible causes during the consultation, particularly when cognitive impairment or limited health literacy were suspected.

### Seamless use of clinical feedback system during consultations

The most common approach to using the CFS in consultations was to integrate it seamlessly as a natural part of the dialogue and the overall consultation process. Unlike their previous practice, the SCNs now had access to information based on the patient’s report. For many, the CFS was embedded in existing routines and interwoven with the various components of the consultation through dialogue, guidance, education, and clinical examination of the ostomy. The CFS introduced both implicit and explicit changes to consultation practice. The SCNs emphasised different aspects of the tool depending on the patient’s needs, their own preferences, and prior experience. As the participants varied in how they structured their consultations, they also conveyed information about the outcomes to patients in a seamless manner, whenever it felt natural. Some explicitly stated during the consultation that they had received and reviewed the patient’s responses. For others, this became apparent more implicitly throughout the different phases of the consultation. Some SCNs consistently followed a fixed sequence for the various components, while others adjusted the structure based on clinical judgement. For the latter group, the order was guided by their understanding of the patient’s challenges, as expressed in the report and through the patient’s descriptions, as well as by what was considered most appropriate in the given situation. One participant emphasised the importance of adapting the structure to the patient’s needs:

*If the patient is sitting there thinking only about leakage*,* and I’ve read in the report that they have a high score*,* we must address the ostomy care and examine it immediately. Otherwise*,* they won’t be able to focus on anything else. It wouldn’t be appropriate to delve into a deeper conversation about something else.*

The SCNs facilitated a more focused and meaningful conversation throughout the rest of the consultation by first addressing what they perceived to be the patient’s most pressing concern. This approach illustrates how the patient report functioned as a source of information and a means of prioritising the consultation content.

A common feature among the SCNs was that the report served as a guide, indicating which topics should be prioritised during the consultation. Further exploration, often triggered by unfavourable scores, was naturally integrated into the dialogue throughout the different phases of the consultation.

The participants prioritised asking open-ended questions about how the patient experienced their situation to encourage open sharing. By beginning with topics that mattered most to the patients, they found that patients became more receptive and motivated to engage in the guidance provided. When patients shifted the conversation towards unrelated topics, several used the CFS as a reference point to steer the discussion back to the most pressing issues. The SCNs used information from the CFS to guide the dialogue towards areas where patients had reported unfavourable scores. This approach was used both with and without visual support from the application:

*When I read your responses*,* I can see that you may be experiencing some challenges with being physically active. Could you tell me more about how that affects you?*

In some cases, the SCNs had already addressed the issues related to unfavourable scores earlier in the consultation, making it unnecessary to revisit them in detail during the final discussion. However, they often referred to the patients’ responses to confirm their understanding:

*I noticed that you scored high on leakage. We’ve already discussed this and decided to try a new ostomy plate.*

Participants were also attentive to clarifying the reasons behind scores related to quality of life, as these could reflect ostomy-specific challenges, other health issues, or aspects of the patient’s overall life situation. They recognised the importance of understanding the patient within the context of their everyday life, while at the same time avoiding prolonged discussions unrelated to ostomy care. Nevertheless, insight into such factors (particularly pain) was essential when assessing the patient’s capacity to absorb new self-care-related information. Although the SCNs often used responses warranting further exploration as a starting point, many emphasised the importance of not overlooking responses that did not initially appear to require further exploration. One participant explained:

*I can’t dismiss a question because the patient scored well. I wonder*,* I’m curious. I ask how they’ve managed to adapt so well. Then*,* things might come up. Maybe it’s not going as well as it seems. I get an intuitive sense when I have the patient in front of me; for example*,* I read their body language and their responses. I compare my current patient observations with their diagnoses and reasons they are here for follow-up.*

This statement illustrates how clinical competence, judgement, and tacit knowledge were applied when SCNs interacted with patients and interpreted their responses from the CFS.

### Shared language and use of visual features

The SCNs learned from experience that using the CFS contributed to developing a shared language between themselves and their patients, thereby facilitating meaningful dialogue during consultations. This shared language was anchored in the patients’ engagement with completing the questionnaire and the SCNs’ prior review and assessment of the responses. Reviewing the responses together, using this shared and mutually understood language, enabled them to explore complex and varied topics.

*Many patients have told me that they’ve acquired a language that makes it easier to express what they mean. When I refer to their responses in the questionnaire*,* they understand better what I’m talking about and vice versa. It makes a big difference. We speak the same language. We create a shared language through the form. Of course*,* I had a language before*,* but it didn’t always match the patient’s language. Now*,* we have a shared reference*,* and I feel more confident we understand one another.*

SCNs further experienced the CFS’s visual features as beneficial, recognising their clinical and pedagogical value; several used them actively during consultations to support dialogue. Some kept the application open on the screen throughout the consultation but primarily used it to summarise the discussion. One participant said:

*I often show the graphs that illustrate the domains of the OAS [Ostomy Adjustment Scale] and the quality-of-life form… they’re easy to understand. I point to them and go through what I see. I explain where the red flags are [indicating low adaptation]*,* what looks good*,* and how this aligns with what we’ve clarified and discussed along the way. Sometimes*,* new things come up when we sit like this. I always ask if the patient has any questions or if there’s something we haven’t touched on.*

Other participants used the application and its visual elements as needed, guided by clinical judgement. The visual representations, particularly the ability to display subscale scores across the different instruments, supported the dialogue by offering a concrete point of reference and an accessible pedagogical tool. The visualisations on screen also served as entry points for discussion, enabling the introduction of new topics or transitions between thematic areas.

*I put a lot of energy into thinking about how to raise different topics with patients*,* especially the more private ones. Having several ways of starting the conversation gives me a certain reassurance. It’s always a matter of situational judgement. No two cases are alike.*

Several SCNs learned that the CFS made it easier to address and explore topics often regarded as sensitive, such as sexuality, body image, or self-esteem. For some, using the CFS represented a shift in clinical practice from a superficial approach to quality-of-life topics towards more in-depth conversations with patients. Nonetheless, they remained attentive to patients’ non-verbal cues and refrained from pursuing topics further if there were signs of discomfort or resistance. However, revisiting such topics in later consultations often led to more open and meaningful conversations. At the same time, some participants perceived the screen as a potential barrier to meaningful interaction. SCNs emphasised the importance of maintaining eye contact and observing body language during consultations and, therefore, chose to postpone using the screen until the summary phase. Additionally, variations in the physical layout of consultation rooms influenced the placement and use of the screen, which in turn affected how visual elements were integrated into the clinical dialogue.

## Discussion

This study is the first to explore how SCNs experience using a CFS in routine outpatient follow-up after stoma surgery. The findings indicate that the CFS was experienced as a valuable tool for promoting personalised and holistic follow-up care. Participants described the CFS as supporting preparation, providing structure for consultations, and enhancing patient involvement. Furthermore, SCNs experienced it useful for mapping, assessing, and evaluating the patient’s adaptation process, particularly in identifying areas of concern. While exercising their clinical judgement, the SCNs described using the tool seamlessly within their existing practice. The development of a shared, intelligible, and harmonised language, together with the CFS’s visual functions, was perceived to facilitate meaningful conversations.

Although perceptions of usefulness varied, participants emphasised that the CFS enabled autonomy and flexibility, allowing them to adapt its use to their consultation style and the patient’s individual needs. Participants also emphasised that it was important for patients to be well informed about the CFS’s purpose of providing feedback through the CFS. Nevertheless, the study revealed considerable variation in how SCNs informed and guided patients, which may reflect differing perceptions of the system’s value. Comparing the findings from patients’ experiences of the same CFS [25], where many did not fully understand its purpose, highlights the need for greater attention to how SCNs inform and prepare patients for its use. Patients’ lack of understanding has also been identified in other clinical settings [40–43] and may influence patient engagement and the perceived usefulness of feedback systems. Further knowledge is therefore needed about how patients can best be informed and prepared, an area for which the literature provides limited guidance [27].

Clinical experience, professional judgement, individual attitudes, and an understanding of the CFS’s purpose appeared to influence how SCNs use the system. Nevertheless, participants consistently described the CFS as supporting preparation, providing structure, maintaining patient focus, and reinforcing patient involvement during consultations. While the latter two functions are consistent with previous studies of CFSs in clinical practice [17], the first offers new insight into how the CFS may support preparation for consultations within a complex healthcare service that has received little attention. These findings are relevant to person-centred practice, where the person rather than the illness is at the centre and where a holistic care, individualisation, and partnership are fundamental [44]. They also illustrate the complexity of using standardised tools into dynamic and complex clinical practice while maintaining professional judgement. Oure findings suggest that use of CFS support SCNs’ in delivering person-centred care, which is the primary aim of its implementation [12].

A noteworthy finding was the development of a shared and mutually understood language using the CFS. Participants described this shared language and system’s visual features facilitating more meaningful, clinical, and person-centred dialogue by reducing linguistic distance between healthcare professionals and patients. Previous studies have similarly shown that feedback system support communication between patients and healthcare professionals [14, 16]. However, introducing a CFSs into practice does not automatically improve interaction [45]. Greenhalgh et al. (2018) argue that such systems influence communication and care practices differently depending on the care context, the system’s structure, and the relationship between the patient and healthcare professional [19]. Our findings further suggest that relationship-building may influence and shape how SCNs use feedback system in clinical practice. Healthcare professionals may perceive such measurement tools as socially situated when their use is closely linked to managing and developing patient relationships [19].

Nevertheless, creating effective communication using a CFS is not a straightforward task and it does not occur automatically, as noted by Mejdahl et al. (2020) [45]. The SCNs in this study particularly valued the visual features, which they experienced as supporting dialogue about complex and sensitive issues, and emotional challenges, issues that often receive limited attention during consultations [4–7]. The practice narratives further suggest that the value of the CFS depends not only on its design but also on how it is used within patient–SCN relationship.

Oure findings suggest that the way the CFS is used constitutes an intervention in itself. Participants described how the tool’s value depended on how it was applied, in which situations it was used, and on the SCN’s ability to interpret and use the feedback reports meaningful. Most SCNs described integrating patients’ responses and the visualisation functions into their existing practice while maintaining flexibility and professional autonomy. This is consistent with Greenhalgh et al. (2018, who highlight flexibility as important for its use [19].

Experience from dialysis care also emphasise the importance of discussing feedback results with patients [46]. Healthcare professionals are encouraged to adopt a guiding role in these conversations, creating a collaborative environment in with patients feel safe to share their experiences. According to patients’ experiences of the same CFS [26], this appears to vary in stoma care consultations, which may reflect differences in SCNs’ attitudes toward the CFS.

Despite these perceived benefits, the study also identified several challenges associated with using the CFS. Some SCNs experienced the system as less valuable, and participants described barriers including limited digital competence and inadequate quality assurance in the distribution of questionnaires. These findings are consistent with previous research showing that limited skills may hinder effective use of feedback systems in clinical practice [20].

An important contextual factor that may have influenced SCNs’ positive experiences of using the CFS was that they themselves initiated its incorporation into clinical practice. This may have fostered a stronger sense of ownership and motivation than is often reported with top-down implementation strategies [21]. Nevertheless, participants described variation in how the CFS was used and in how well it fitted into their routine workflow.

Participants also emphasised the importance of training and peer support in strengthening their confidence and competence in using the CFS. Our findings suggest that the perceived value of PROs depended on their active use during consultations through discussion and shared reflection. This is consistent with recommendations that healthcare professionals actively share and discuss PROs in routine practice [16]. Anderson et al. [47] similarly identify professional training as an important prerequisite for sustainable implementation. Together, these findings suggest the importance of strengthening digital competence, providing systematic training and guidance, and supporting reflection on how patient-reported information is communicated and used during clinical consultations [47, 48].

This study extends previous research on the use of PROs by providing insight into how SCNs experience using a CFS in routine ostomy care. Participants perceived the system as particularly relevant in a clinical context where physical, psychosocial, and social adaptation are central [24]. The findings further suggest that the use of a CFS is shaped by professional judgement, clinical context, and the relationship between the SCN and the patient, rather than by the system alone. When used thoughtfully and adapted to the individual consultation, participants experienced the CFS as supporting person-centred care and clinical practice.

### Strengths and limitations

Including perspectives of all SCNs, irrespective of their employment percentage or level of experience with CFSs, represents a strength of this study. The use of visual flowcharts facilitated the elicitation of rich and varied practice narratives, illuminating multiple dimensions of the research question. We have provided a detailed account of the analytical process thereby enabling readers to follow the pathway from data to conclusions [37]. However, retrospective accounts may not fully capture Participants’ immediate experiences, representing an inherent limitation [49]. To generate more detailed and actionable insights into how the CFS is used in practice, it is necessary to employ additional methods, such as fieldwork, that go beyond what conventional interviews can offer. As the study was conducted within a Norwegian healthcare context with specific organisational structures, readers should consider the transferability of the findings to other healthcare systems cautiously. Nonetheless, our findings are valuable and further extend existing contributions to the clinical feedback literature.

### Relevance to clinical practice and further research

Notably, the study underscores the emergence of a forward-looking nursing role in which digital tools are integrated into everyday clinical routines to enhance the delivery of personalised care. By acknowledging and building upon these established practices, healthcare services can advance a more standardised and supportive use of CFSs. Developing local procedures, encouraging peer learning, and facilitating reflective discussions among SCNs appear to be central to deeply embedding such tools in clinical workflows, thereby contributing to improved continuity and quality of ostomy care.

To support effective use of the CFS in clinical practice, it is essential to develop structured training programs and to acquire educational resources for SCNs. Given these requirements, the study identified several distinct ways in which PROs are operationalised in clinical practice. These practical applications offer a valuable foundation for designing targeted educational initiatives and implementation strategies, thereby reinforcing the contribution of measurement-based follow-up to routine ostomy care. Further research is warranted to inform the development, content, delivery, and effects of these training programs. This includes the application of appropriate qualitative methodologies to gain a deeper understanding of how the intervention is used and integrated into clinical workflows, how a CFS shapes nurse-patient interactions, and which specific content areas and competencies the training should address.

## Conclusion

This study illuminated how SCNs experienced using a CFS in routine ostomy follow-up care. Through the SCNs’ narratives, the study showed that they experienced the CFS as more than a tool for assessing patient function and adaptation and described how it shaped the way they conducted follow-up consultations. The SCNs perceived that meaningful use and integration of the CFS into routine clinical practice supported their work in tailoring holistic follow-up care to individual patients. They experienced the use as a structured aid that contributed to more reflective, consistent, and patient-centred clinical conversations. These findings suggest that the use of CFS in routine ostomy care has the potential to facilitate person-centred dialogue and support person-centred care.

## Supplementary Information

Below is the link to the electronic supplementary material.

Supplementary Material 1

Supplementary Material 2

Supplementary Material 3

Supplementary Material 4

## Data Availability

The dataset supporting this study’s findings is not openly available due to privacy reasons, but it is available from the corresponding author upon reasonable request.

## Declarations

### Patient and public involvement

Three patient representatives, recruited by Førde Hospital Trust, were involved in the development and refinement of the interview guide.

## Abbreviations

SCN: Stoma care nurse
PRO: Patient reported outcome
PROM: Patient reported outcome measures
CFS: Clinical feedback system
