# Supplementary material for: Stoma care nurses’ experiences using a clinical feedback system in routine outpatient follow-up: a qualitative study
Source: BMC Nurs. 2026 Jul 22;25:988. doi: 10.1186/s12912-026-05100-1 (PMC13673585; doi:10.1186/s12912-026-05100-1)
Supplement: Supplementary file 1 [file 12912_2026_5100_MOESM1_ESM.docx]

Interview guide: Stoma care nurses’ experiences with a clinical feedback system

## The Professional

- Could you tell me a bit about your professional background and what your daily work responsibilities entail?
- What were your initial thoughts when the clinical feedback system became a topic at your workplace?
- Can you describe your role and experience with the process of developing and implementing the clinical feedback system?
- How has the training in using the system been for you?
- From your perspective, what is the purpose of using the clinical feedback system?

## PAtient patHway

- Can you describe how you follow up with patients in your practice? Feel free to use a patient you are currently following up as an example.
- Do you have different experiences with patients in your practice?
- What do you emphasize most in the follow-up and how do you do this?
- Can you describe what you do to adapt available support to the patient’s needs and goals?

## practice and General questions

- Can you describe how the clinical feedback system functions in the patient’s follow-up.
- What significance does the clinical feedback system have for you?
- Has the clinical feedback system changed how you follow up with patients? – How? Please elaborate.
- How have you incorporated the clinical feedback system into your way of working?
- How well do you think the purpose of the clinical feedback system aligns with how it works in practice?
- What is your impression of how patients experience the feedback system?
- What is your impression of how your colleagues experience using it in daily work?

## Summary

- If you were to highlight the most important aspect of using the clinical feedback system, what would it be?
- From your perspective, what changes or adjustments are necessary to increase the potential usefulness of the clinical feedback system as part of the follow-up for both you and the patients?
- Do you always use the questionnaires for the patients you follow up with? (Why or why not?
- Is there anything we haven’t talked about or anything you haven’t had the chance to share?
- Do you have any questions?

## Post-interview question

- How did you experience our conversation today?

Follow-up questions if relevant information does not emerge in the practice narratives

## Preparation

- How do you inform patients about the use of the feedback system? What do you say?
- How do you prepare for patient consultations?
- Can you describe how you proceed when you are assessing the information you receive?
- What is different about the current way of preparation compared to the way of preparation before using the clinical feedback system?

## consultation and communication/relationship

- Can you describe how the consultation is conducted?
- What do you find helpful or unhelpful about the clinical feedback system during the consultation?
- How do you feel the clinical feedback system influences communication?
- What is it like to talk with patients using a clinical feedback system versus no feedback system?
- How do you assess the need for further follow-up?
- How is the consultation concluded?

## After the consultation

- Describe the tasks and assessments you conduct after the consultation.

## Personalized healthcare and User involvement/shared decision-making

- What do you emphasize most in the follow-up and how do you manage it?
- What role does the feedback system play concerning individual needs and goals?
- How do you proceed in practice?
- How do you experience the effects on patient’s involvement after using the feedback system?
- Has anything changed about making treatment or care decisions when using the clinical feedback system?

## competence

- What skills are important in how you use the clinical feedback system?
- How do you assess your skills in using the clinical feedback system?
- How do you assess or use clinical judgment with the feedback system?
- How do you develop your skills in using the clinical feedback system? What will be important for you moving forward?

## quality of care

- What do you emphasize to ensure high quality in your work? How?
- What does your use of the clinical feedback system mean for quality of care?
