# Supplementary material for: Stoma care nurses’ experiences using a clinical feedback system in routine outpatient follow-up: a qualitative study
Source: BMC Nurs. 2026 Jul 22;25:988. doi: 10.1186/s12912-026-05100-1 (PMC13673585; doi:10.1186/s12912-026-05100-1)
Supplement: Supplementary file 2 [file 12912_2026_5100_MOESM2_ESM.docx]

First contact

Prior concultation

During consultation

After concultation

The figure served as a visual aid to remind participants of the sequential steps in the follow-up process used during interviews.
