# Supplementary material for: Stoma care nurses’ experiences using a clinical feedback system in routine outpatient follow-up: a qualitative study
Source: BMC Nurs. 2026 Jul 22;25:988. doi: 10.1186/s12912-026-05100-1 (PMC13673585; doi:10.1186/s12912-026-05100-1)
Supplement: Supplementary file 3 [file 12912_2026_5100_MOESM3_ESM.docx]

Ostomy Adjustment Scale

Health-related Quality of Life

Clinical form

The figure presents examples of how patient-reported outcomes are visualised for the Ostomy Adjustment Scale, the COOP-WONCA chart, and a clinical form, serving as a visual aid to remind participants during data interviews.
