# Supplementary material for: Stoma care nurses’ experiences using a clinical feedback system in routine outpatient follow-up: a qualitative study
Source: BMC Nurs. 2026 Jul 22;25:988. doi: 10.1186/s12912-026-05100-1 (PMC13673585; doi:10.1186/s12912-026-05100-1)
Supplement: Supplementary file 4 [file 12912_2026_5100_MOESM4_ESM.docx]

# Analyses process: Reflexive thematic analysis

A six-phase reflexive thematic analysis was undertaken to explore patterns across the dataset. Although the six phases are conceptually distinct, the process is recursive, requiring repeated movement between phases in a spirit of inquiry and ongoing reflection. LAJ led the analysis in collaboration with AMS, while JRA contributed at various stages to provide alternative perspectives and deepen the analytic interpretation.

## Phase 1: Familiarizing ourselves with the dataset

LAJ conducted and transcribed all the interviews. The process of transcribing the interviews provided an opportunity to become thoroughly familiar with the dataset. LAJ assessed the dataset to determine its potential to address the research question (RQ): *What are the experiences of stoma care nurses with a clinical feedback system in routine outpatient follow-up care?* Repeated reading of hard copies and listening to the audio files throughout the analytical process revealed that moving between different media prompted new reflections, insights, and interpretations. Significant segments of the data were marked in the audio files and in the margins of the transcripts. Brief analytic notes were written to capture reflections and ideas about specific parts of the data and the dataset as a whole. Brief analytic notes were written to capture reflections and ideas about specific parts of the data and the dataset as a whole. Initials analytic observations and interpretations were primarily inductive, with attention directed towards the latent meanings underlying participants' accounts.

## Phase 2: Coding

LAJ then moved into a more systematic and rigorous second phase of the analysis, working through the entire dataset to identify segments relevant to the research question and applying analytically meaningful codes in an inclusive and comprehensive manner. The codes encompassed both semantic and latent meanings, reflecting LAJ's analytical interpretation of the data. Data extracts were cut from the transcripts, mounted on file cards, and annotated with a code label and participant number. During coding, the codes evolved as engagement with the dataset deepened. Codes were expanded, split, merged, refined, and renamed to better capture participants' meanings. For example, initial codes such as *information conveyed to patients*, *information content*, *preparation content*, and *emphasis on information* recurred across the dataset and were progressively refined throughout the coding process.

## Phases 3: Generating initial themes

Initial themes were generated by identifying shared patterns of meaning across the dataset. This active process, led by LAJ in collaboration with AMS, involved clustering codes based on their similarities to generate initial themes. A large wall was used to map out potential clusters, with file cards attached to the wall and rearranged throughout the process. The relationships between the developing initial themes were reviewed, and the coded data were systematically reviewed against the dataset to support the identification of potential themes. At this stage, five initial themes were identified. The example codes presented in the previous phase were among those that informed the generation of an initial theme titled *A New Way of Preparing for Consultations.*

## Phase 4: Developing and reviewing themes

In this phase, the quality of the themes and the boundaries between them were assessed. It became apparent that the themes were overly diverse and wide-ranging, lacking the necessary focus. They encompassed both experiences related to the use of the system and issues concerning its implementation. As a result, they failed to capture the overarching narrative about the entire dataset and the RQ. The initial RQ was therefore refined to focus more specifically on experiences with using the clinical feedback system in clinical practice: *What are the stoma care nurses' experiences using a clinical feedback system in routine outpatient follow-ups?*

Consequently, LAJ undertook additional coding (Phase 2), set aside the data material related to implementation, and revisited Phase 3 to regenerate the initial themes relevant to the revised research question. This process resulted in one overarching theme and three initial themes. Upon returning to this phase, we ensured that the themes were aligned with the extracted codes and the full dataset before finalizing the thematic map.

RQ: Experiences using a clinical feedback system Overarching theme: A tool for person-centred care and holistic consultation

New way of preparing for consultation

Shared language and use of visual features

Various approaches in consultation

Flexible use of clinical feedback system during consultations

**Fig. 1.** Overarching themes with initial codes from Phase 4.

## Phase 5: refining, defining and naming themes

By writing short abstracts of the themes, we ensured that they became demarcated, constructed around robust core concepts, and integrated into the overall narrative about the data. LAJ opted to revisit four initial themes for further review, which involved moving back and forth between the phases. This process led to the merging of two themes to establish a core concept: *The flexible use of a clinical feedback system during consultation* and *Various approaches in consultation* were merged into a new concept, named *Seamless use of CFS during consultation*. As a result of this phase, we identified three themes that addressed the RQ. Informative names were selected for each theme, resulting in the following themes: (1) *Providing and Receiving Information as Preparation for Consultations*, (2) *Seamless Use of the Clinical Feedback System During Consultations*, and (3) *Shared Language and Use of Visual Features*. The overarching theme was retained.

## Phase 6: Write-ups

Guided by an abstract from each previous phase, we composed the analytic narrative using relevant empirical extracts from the entire dataset. This was an integral part of the analytic process, involving extensive writing and rewriting in collaboration with the research group and service users. This phase consisted of weaving together our analytic commentary and our data extracts, which were organised and presented through our themes.

We selected vivid and compelling data extracts to illustrate each theme and were mindful of demonstrating patterns across the dataset rather than relying on isolated examples. Some participants articulated their experiences particularly vividly and evocatively. Therefore, we were attentive to selecting extracts from different participants and different data items to illustrate each theme. Although extracts from most participants were included, ethical considerations related to the possibility that participants might recognise one another meant that participant numbers were not reported alongside the quotations.
